# Characterisation of survivability resilience with dynamic stock interdependence in financial networks

**DOI:** 10.1007/s41109-018-0086-z

**Published:** 2018-07-31

**Authors:** Junqing Tang, Layla Khoja, Hans R. Heinimann

**Affiliations:** ETH Zurich, Future Resilient Systems, Singapore-ETH Centre, 1 CREATE Way, CREATE Tower, Singapore, 138602 Singapore

**Keywords:** Survivability resilience, Financial stock networks, Network dynamic evolution, Weighted mtultinomial logistic regression

## Abstract

This paper examines the dynamic evolutionary process in the London Stock Exchange and uses network statistical measures to model the resilience of stock. A large historical dataset of companies was collected over 40 years (1977-2017) and conceptualised into weighted, temporally evolving and signed networks using correlation-based interdependences. Our results revealed a “fission-fusion” market growth in network topologies, which indicated the dynamic and complex characteristics of its evolutionary process. In addition, our regression and modelling results offer insights for construction a “characterisation tool” which can be used to predict stocks that have delisted and continuing performance relatively well, but were less adequate for stocks with normal performance. Moreover, the analysis of deviance suggested that the survivability resilience could be described and approximated by degree-related centrality measures. This study introduces a novel alternative for looking at the bankruptcy in the stock market and is potentially helpful for shareholders, decision- and policy-makers.

## Introduction

Complex network approaches are commonly applied in a wide range of academic fields (Barabási 2016; Münnix et al. 2012), and studies on network topology have always been an interesting topic. The statistical measures of topology and interdependence are often strongly associated with the performance of network components. For example, in financial stock networks, the correlation-based topology varies with the different condition of nodes (stocks) and edges (correlation-based interdependence). Here, we investigated such associations and tried to establish a predictive relationship between network measures and a special type of component performance, survivability resilience, in correlation-based stock networks.

As self-explained, the term survivability resilience describes the ability of the subject to survive, to be reliable, and to avoid failure in the environment (Singpurwalla 1995). It answers the question of how resilient the subject is in a static or dynamic environment to maintain long survival (Sterbenz et al. 2010). In the stock market, the survivability is termed to illustrate the ability of stocks/listed firms to prevent corporate failure/bankruptcy or being delisted from the market. Categorising and predicting corporate failure is essential in bankruptcy studies (Khoja et al. 2016) because it is of great importance in providing early warnings about a company’s financial distress to stakeholders, business managers, policy-makers, and financial economists (Amendola et al. 2017; Jones et al. 2017) and it is hard to be characterised and predicted (Allen and Babus 2009).

Over the past 50 years, various statistical models based on financial or accounting data have been applied for predicting corporate bankruptcy (du Jardin et al. 2017). The most frequently used methods for studying stock survivability include genetic fuzzy models (Kuo et al. 2001), artificial networks (Zhang et al. 1999), genetic algorithms (Zelenkov et al. 2017), and neural networks and deep learning networks (Ticknor 2013; Chong et al. 2017). Other traditional statistical models have also been proposed, such as multivariate discriminant analysis and logistic regression (Beaver 1966; Altman 1968; Shumway 2001; Mossman et al. 1998; Lee et al. 1996). In recent years, machine learning models have been popular in bankruptcy predictions due to their excellent performance on accuracy (Barboza et al. 2017). However, the majority of those models require a substantial amount of accounting-related data (from a company’s financial statement) as input variables (du Jardin et al. 2017). This sometimes leads to an unpromising issue as those accounting data could not always be available in hand. Furthermore, most studies of bankruptcy have concentrated on only failed firms and overlooked the possibility of using networks perspectives to model stocks/firms with different survivability, including those of exceptionally resilient performance.

On the other hand, apart from bankruptcy literature, studies of financial stock networks themselves are not rare in the literature. One interesting topic is to study the temporal transformation of the market with a network perspective. The special swarm patterns caused by different stock’s survivability performance often manifest in the network evolution process. However, most of the previous works have only briefly discussed such process of their studied networks (Bonanno et al. 2004; Mantegna 1999; Onnela et al. 2003), and most have been based only on either a short time period or a small fraction of the market population (Huang et al. 2009; Gao et al. 2013). We believe that studying the long-term evolutionary process of the market networks would help us understand more about the survivability resilience of stocks.

Thus, the two-fold purposes of this paper are: (a) firstly, to explore the correlation-based interdependence of a whole market by constructing weighted, signed and temporal stock networks and to understand the long-term dynamic evolution of their topological features; and (b) with the understandings of long-term historical evolution process from the first purpose, we then characterise the survivability resilience of stocks via statistical models (using interdependence and network measures as variables) and then explore their predictive strengths by identifying highly descriptive parameters. This work is an expanded version of preliminary work in (Tang et al. 2017). Here we expanded the scopes by using a new and more completed dataset and applying new modelling approaches. Also, we tested and validated the predictability of the model and studied its performance regarding various stock behaviours.

The remainder of this paper is organised as follows: “Data and methodology” section describes the data and methodology for network construction, followed by analysis of the dynamic evolving process in “Understanding interdependence” section. In “Time-series network measuresTime-seriesnetwork measures” section, six network measures are introduced and their statistical analysis are presented. “Survivability and resilience characterisation” section consists detailed results and discussion on survivability resilience, followed by final conclusions summarised in “Conclusion” section.

## Data and methodology

We used DataStream ^*TM*^ to gather historical data on the daily closing stock prices (adjusted stock price, which accounts for actions such as splits and dividends) for 7206 companies that had ever traded or were still trading on the London Stock Exchange over a 40-year period (total of 10438 trading days), from 04/05/1977 to 05/05/2017.

Firstly, we categorised all stocks before constructing networks. The categorisation of Delisted companies and Continuing companies were based upon their ability to survive in the markets. Stocks that did not belong to either of those two groups were treated as Normal companies. The following definitions were used for our categories:

- **Delisted stocks** (example stocks 1 and 2 in Table 1): those companies that were delisted when they have a high leverage generally because they were unprofitable, and/or were facing difficulties in gaining additional equity capital during their public life (Pour and Lasfer 2013). Consequently, those companies have been delisted to become privately owned companies, acquired companies or in some cases, went bankrupt.

**Table 1:** Structures for three groups of collected data

Yearly window	Date	stock 1	stock 2	stock 3	stock 4	stock 5	stock 6
Year 1	date 1	232.2	-	732.5	101.0	-	-
	date 2	186.2	-	232.2	106.9	-	-
	date 3	148.7	162.9	186.2	1026.7	-	-
	…	112.7	168.2	148.7	218.8	-	-
…	…	82.2	185.0	112.7	732.8	-	-
Year 10	…	82.2	185.0	112.7	732.8	-	-
	date 100	59.7	108.4	82.2	232.3	-	-
	date 101	82.7	121.2	59.7	186.3	232.2	-
	date 102	163.8	201.0	82.7	148.8	186.2	-
	…	154.8	154.8	163.8	112.8	148.7	2.6
…	…	154.8	154.8	163.8	112.8	148.7	2.6
Year 25	…	108.1	-	154.8	82.2	112.7	2.7
	date 10000	95.4	-	108.1	59.7	82.2	2.8
	date 10001	-	-	59.7	82.8	59.7	1.4
		-	-	82.7	163.9	82.7	1.0
…	…	-	-	82.7	163.9	82.7	1.0
Year 40	…	-	-	82.7	163.9	82.7	1.0
	date 10437	-	-	163.8	154.8	163.8	2.1
	date 10438	-	-	154.8	108.1	154.8	2.9

The “Date” column indicates the number of trading days into the 40-year observation period. Stocks 1 and 2 illustrate the Delisted group, stocks 3 and 4 are examples of the Continuing group, and stocks 5 and 6 represent companies in the Normal group. In each column, values are the average closing prices for that date
- **Continuing stocks** (example stocks 3 and 4 in Table 1): those companies have good opportunities for investment growth, and which showed increases in equity capital when quoted in the market (Pour and Lasfer 2013). For our purpose, continuing group represents the companies which have been continuing to trade in the market for the entire 40-years observation period.
- **Normal stocks** (example stocks 5 and 6 in Table 1): those companies were initially listed at some point during the observation period and had not failed yet by the end of the observation period.

Next, we determined the edges of these complex financial networks, based on predefined interdependence that characterised a certain relationship or interaction between acting nodes. A considerable number of studies have focused on methods for constructing the edges in stock networks. They include the minimal spanning tree (Bonanno et al. 2003; Vandewalle et al. 2001; Kwapień et al. 2017), planar maximally filtered graph (Tumminello et al. 2005), threshold filtering mechanism (Huang et al. 2009), and winner-takes-all approach (Chi et al. 2010). Other more recent investigations have concentrated on the methods for constructing interdependence, e.g., Pearson correlation coefficients (Heiberger 2014), Partial correlation coefficients (Xu et al. 2017), Pearson product-moment correlation coefficient (Zhang et al. 2017), covariance and Gaussian graphical models (Xuan and Murphy 2007). Generally speaking, the Pearson correlation coefficient tends to be the most widely applied methods.

Therefore in our study, we used the Pearson correlation coefficients to construct networks, using pair-wise logarithmic returns for stocks on a daily basis. For this, we let *r*_*i*_(*t*) and *p*_*i*_(*t*) denote the log-return and closing price of stock *i* at time *t*, respectively. The daily log-return can be expressed as follows:

1 $$ r_{i}(t)=ln\left[p_{i}(t)\right]-ln\left[p_{i}(t-\Delta t)\right]  $$

where *Δt* is one trading day, *Δt*=1. Then we write Pearson correlation coefficients (Benesty et al. 2009) *c*_*i*,*j*_ between stock *i* and *j* as:

2 $$ c_{i,j}=\frac{<r_{i}(t)\times r_{j}(t)>-<r_{i}(t)>\times <r_{j}(t)>}{\sigma_{i}\times \sigma_{j}}   $$

where <.> indicates the mean value and *σ*_*i*_ is the standard deviation of the stock *i* in a time series. The *p*-values were also computed for each coefficient and used as the threshold to prune the networks and filter out those insignificant correlations. In order to avoid severe topological information loss while pruning the edges (according to the evidence shown in Huang et al. (2009), the edge density of stock network drops sharply from *c*_*i,j*_=0.1), we set *p*-value threshold as 0.01 to eliminate weak correlations for − 0.1<*c*_*i,j*_<0.1, replacing them with “0”. We then used the coefficient values as edge weights to represent the intensity of connections. Like the positive/negative interactions in social networks (Leskovec et al. 2010), we also showed considerations to negative signs in the correlation-based financial networks, and the edge signs were same as the corresponding signs of those coefficients.

In the final step, networks were constructed based on the yearly time window, which resulted in 40 networks in total (c.f., Table 1). One should be aware of that we need to identify the population of active stocks in each constructing year. For example, the stock 5 in the table cannot be included until year 10 since it was not listed during those years. However, if a particular stock was newly de-listed in the middle of a given year, e.g., stock 1 in Year 25, it was still considered active for that year because some closing price records remained available in that specific yearly window. It was only counted as inactive thereafter. Thus, for all active stocks in one year, the correlation coefficients were calculated in a “pairwise” manner, meaning that if one of the two columns contained a series of value “NAN” from a certain row, all rows with value “NAN” were omitted and only the common section was used to calculate the coefficient.

## Understanding interdependence

In this section, we investigate the basic network information extracted from the stock networks and study the dynamic evolution of correlation-based interdependence in long-term observation.

### Network topology

**Fig. 1:**
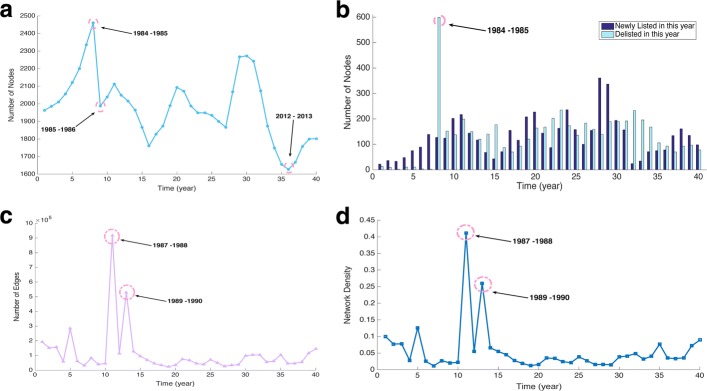
Evolution of network attributes in time. **a** number of nodes, **b** number of newly listed and delisted nodes, **c** number of edges and **d** density of the network

The growth of networks shows a constant fluctuation in terms of the total number of nodes (Fig. 1a), the number of newly listed and delisted nodes (Fig. 1b), the number of edges (Fig. 1c), and the network density (Fig. 1d). Counterintuitively, the networks did not evolve constantly as the market population gradually increased. Subplot (a) presents three major shrinkages and expansions of the market population (Table 2). The first continuous increment occurred during the first eight years when the number of total nodes increased from 1963 to 2336. However, between 1984 and 1986, numerous stocks (599) were de-listed due to a severe recession in the UK in the early 1980s. This was followed by an increasing number of bankruptcy cases (Rhim 1993).

**Table 2:** Statistic summary of 40 constructed networks

No.	Year	Number of nodes	Number of edges	Mean degree	Density	Newly listed	Delisted	Net growth
1	1977-78	1963	192913	196.5	0.1002	22	13	9
2	1978-79	1986	152314	153.4	0.0773	36	9	27
3	1979-80	2010	157144	156.4	0.0778	33	2	31
4	1980-81	2056	58959	57.4	0.0279	48	10	38
5	1981-82	2121	283672	267.5	0.1262	75	10	65
6	1982-83	2200	60651	55.1	0.0251	89	3	86
7	1983-84	2336	32044	27.4	0.0117	139	2	137
8	1984-85	2461	82441	67.0	0.0272	127	599	-472
9	1985-86	1988	40221	40.5	0.0204	124	152	-28
10	1986-87	2039	45687	44.8	0.0220	202	138	64
11	1987-88	2113	917427	868.4	0.4112	217	199	18
12	1988-89	2049	114460	111.7	0.0546	144	150	-6
13	1989-90	2016	528287	524.1	0.2601	117	120	-3
14	1990-91	1964	127319	129.7	0.0660	68	140	-72
15	1991-92	1867	95383	102.2	0.0548	43	177	-134
16	1992-93	1760	70120	79.7	0.0453	71	87	-16
17	1993-94	1828	45818	50.1	0.0274	155	70	85
18	1994-95	1874	33363	35.6	0.0190	116	92	24
19	1995-96	1989	23959	24.1	0.0121	208	120	88
20	1996-97	2093	34956	33.4	0.0160	227	164	63
21	1997-98	2072	75287	72.7	0.0351	144	168	-24
22	1998-99	1988	67088	67.5	0.0340	87	202	-115
23	1999-00	1949	46248	47.5	0.0244	163	235	-72
24	2000-01	1949	39917	41.0	0.0210	236	173	63
25	2001-02	1934	72085	74.5	0.0386	158	135	23
26	2002-03	1900	49091	51.7	0.0272	100	183	-83
27	2003-04	1867	26718	28.6	0.0153	155	159	-4
28	2004-05	2069	34537	33.4	0.0161	361	139	222
29	2005-06	2267	37860	33.4	0.0147	337	189	148
30	2006-07	2273	98924	87.0	0.0383	194	189	5
31	2007-08	2242	103821	92.6	0.0413	157	192	-35
32	2008-09	2073	104141	100.5	0.0485	24	233	-209
33	2009-10	1874	55845	59.6	0.0318	34	196	-162
34	2010-11	1749	62130	71.0	0.0406	71	168	-97
35	2011-12	1656	105045	126.9	0.0767	75	106	-31
36	2012-13	1627	46304	56.9	0.0350	78	93	-15
37	2013-14	1667	46774	56.1	0.0337	134	70	64
38	2014-15	1758	55231	62.8	0.0358	161	92	69
39	2015-16	1800	116737	129.7	0.0721	135	96	39
40	2016-17	1802	145024	161.0	0.0894	98	78	20

The second expansion was found in 1992 to 1993 (16th year), when the market grew from 1760 stocks to 2093 in 1996-1997 (20th year), after that the number gradually decreased again until 2003-2004. In the following two years (28th, 2004-2005 and 29th, 2005-2006), the market rapidly expanded. However, from the 30th year (2006-2007), the market rapidly downsized to 1627 stocks in 2012-2013. This trend is even more apparent in subplot (b), which shows the rise and fall in the number of newly listed and delisted stocks. It is interesting that a major network synchronisation existed in the number of edges (see subplot (c)), where a dramatic change in the number of nodes did not necessarily lead to a similar change in the number of edges. This synchronisation during a period of massive shrinkage might have, in fact, improved the correlations between stock pairs, possibly leading to a slight change in the number of edges. This is also manifested in the density measure in subplot (d), where the network appeared to evolve with same-shape fluctuations. These static measures were strongly associated with the distribution and number of edges, indicating a dynamic shrinking-and-expanding behaviour in network sparsity and topology. This could have been a set of responses by the market to external stimuli that resulted in a “fission-fusion” evolving behaviour.

### Visualisation and basic features of dynamic evolution

We used *Gephi* with Fruchterman Reingold layout algorithm (Fruchterman and Reingold 1991) to visualise eight networks that roughly maintained an equal time gap. This algorithm is a famous member of a force-directed family, utilises nodes that are symbolised as solid objects and the edges acting as “springs” between them. By minimising the energy of the system, the algorithm moves the nodes and changes the forces between them until finally achieves an equilibrium state and then terminates.

**Fig. 2:**
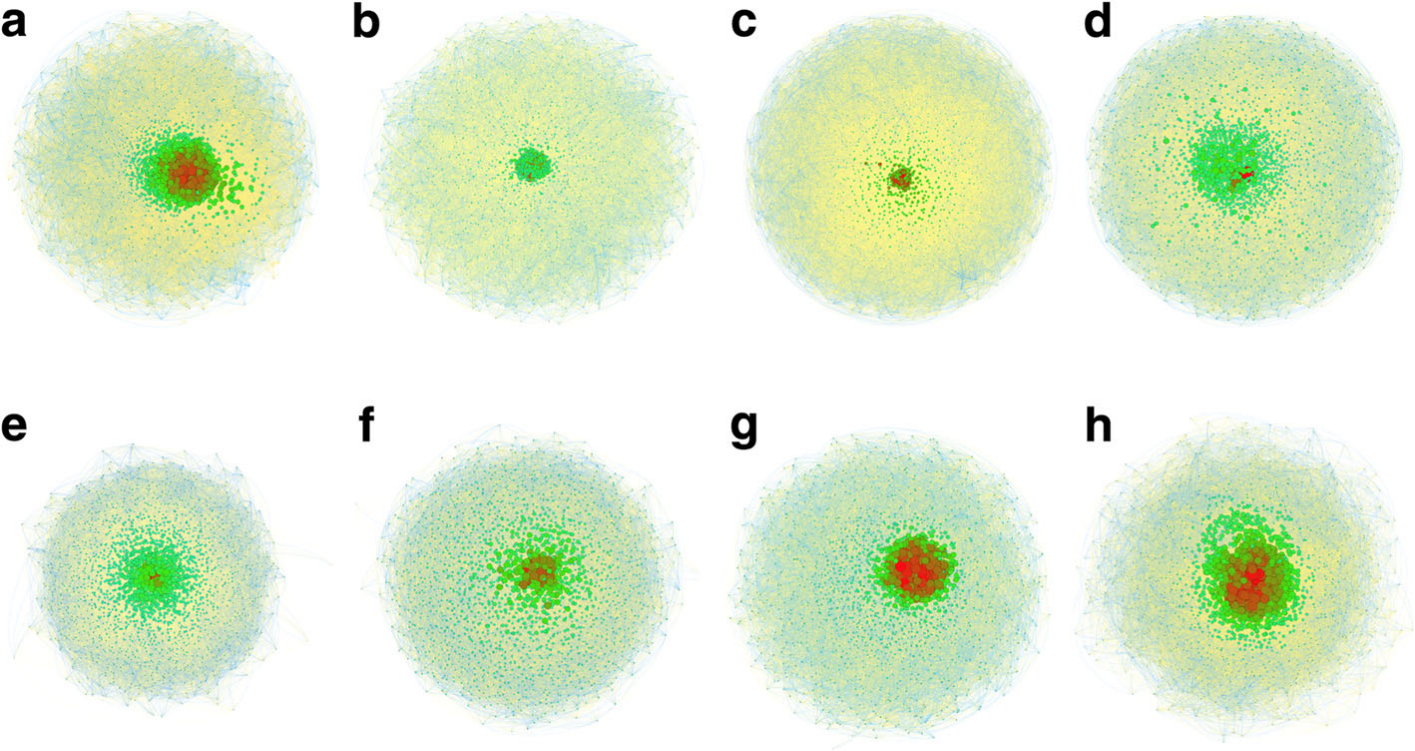
Dynamic evolution of companies in London Stock Exchange from 1977 to 2017, depicting small-world effect in networks at each step. Node color and size: large red, high degree of centrality; small blue, low degree. Edge color: yellow, positive edge; light-blue, negative edge. Thickness of edges is proportional to weight. **a** 1977-1978; **b** 1983-1984; **c** 1988-1989; **d** 1993-1994; **e** 1997-1998; **f** 2003-2004; **g** 2009-2010; and **h** 2016-2017

Figure 2 shows the visualisation results for eight selected networks with an “atom-like” structure, wherein a few nodes were highly interconnected while the rest were sparsely connected around the core. Here, the color and the size of the nodes corresponded to their degree centrality, ranging from large red (high degree) via medium-green to small blue (low degree). Positive edges (positive correlation/interdependence) were indicated with yellow, and negative ones (negative correlation/interdependence), with light-blue. The thickness of the edges was proportional to their weights.

As can be seen, several high-degree nodes formed a core in each network, which indicated an uneven distribution of edges, i.e., nodes in the core area have a high tendency to connect with other high-degree nodes while nodes with fewer connections were more likely to be marginalised. We also determined that the core area (highly interconnected stocks) changed in size, possibly due to the “fission-fusion” evolving behaviour, which denotes a dynamic and unstable picture of interdependence among stocks.

In addition, most of the positive edges were concentrated around the core area while the negative edges were positioned toward the periphery, such as in subplots (a), (c), (d), (e), and (h). This interesting distribution of edge signs indicates that, in some years, the core stocks play influential roles as they not only positively interdependent with each other, but also have positive connections with other marginalised stocks. However, it also can be observed that this intriguing pattern does not stably last throughout the time. For example, in subplots (b), (f), and (g), it is difficult to observe aforementioned clear polarisation on the distribution of positive and negative edges around the core.

Table 3 shows some basic features of the corresponding networks. The small diameters (most of the networks have a diameter no greater than four) and small average path lengths (less than three) again verify a highly interactive and interdependent feature of the stock networks, which in addition denote a “small-world” effect. Taking a closer look at the percentage of edge signs in each network, we found that the ratio of positive and negative edges can be, although with fluctuations, approximated as 9:1, which indicated that a large number of the interdependence between stock pairs was positively correlated based on our network construction method. Such a high percentage of positive correlations could be one of the consequences of simultaneous market synchronisation under market crisis (Kauê Dal’Maso Peron et al. 2012) (N.B. Because various methods exist for constructing correlation coefficient matrix, the pattern we observed here is inferred by applying Pearson correlation method. In other cases, such as using excess returns with Partial correlation coefficients, the percentage and distribution of the edge sign would be different, see an illustrative example of the year 2016-2017 in Appendix 1. However, the comparative study on various methods is beyond the scope of this study. The interested readers can refer to Baba and Sibuya (2005); Kenett et al. (2010)).

**Table 3:** Network statistics of illustrated networks

Visualisation	Year	Diameter	Average path length	Positive edges	Negative edges
a	1977-1978	3	1.848	97.91%	2.09%
b	1983-1984	4	2.129	89.72%	10.28%
c	1988-1989	3	1.955	96.71%	3.29%
d	1993-1994	3	2.013	89.36%	10.64%
e	1997-1998	3	1.958	92.58%	7.42%
f	2003-2004	3	2.175	82.29%	17.71%
g	2009-2010	4	2.152	83.61%	16.39%
h	2016-2017	4	2.013	78.63%	21.37%

## Time-series network measures

Based on the understandings of the interdependence and features of topology evolution obtained from the previous sections, we then investigated the possibility of using more detailed network measures to characterise stocks with different survivability performance. In this way, we could determine which network measures could differentiate the stocks among different performance groups. Here, we excluded the flow- and route-oriented network measures, such as betweenness centrality and closeness centrality, because the flow and route choice are not issues in correlation-based networks.

The six selected network measures chosen for our review were: (1) Degree, *k*; (2) Strength, *s*; (3) Negative degree, *k*^−^; (4) Eigenvector centrality, *e*; (5) Clustering coefficient (CC), *c*; and (6) Average neighbour degree (Ave. neighbour. degree), *x*. The selection criteria were based on the consideration of their popularity and universality in network literature. We also paid particular attention not only to the interdependence of a target node, but also to the condition of its neighbour nodes as well (i.e., eigenvector centrality, CC, and Ave. neighbour. degree). Here, we briefly explain them as follows (for interested readers, more details can be found Barabási (2016); Erciyes (2014); Newman (2010)).

1. **Node degree** is a straightforward nodal measure in complex networks, providing an indication of the importance of the node in terms of the number of its neighbours. For an undirected network of *n* nodes, the degree *k*_*i*_ of node *i* can be expressed in an adjacency matrix as:
  3 $$ k_{i}=\sum\limits_{j}^{n} A_{ij}  $$
2. Yook et al. (2001) and Barrat et al. (2008) have studied the **Node strength** *s*_*i*_ of network properties in weighted networks. This measure assesses the importance of a particular node in terms of its connection intensity. Node strength is defined as the sum of the weights on its total connections/degree. Let *W*_*ij*_ denotes the edge weight matrix corresponding to adjacency matrix *A*_*ij*_, the strength *s*_*i*_ can be expressed as:
  4 $$ s_{i}=\sum\limits_{n}^{j}W_{ij}  $$
3. In general, most existing network studies simply encode whether interdependence exist or not (Chiang et al. 2014). The sign of the interdependence is normally neglected for topological simplification. However, the nodes with a large portion of negative interdependence might have some characters that of great interests for understanding the special features such as hidden community clusters (Ma and Zhang 2018) and structure balance (Anchuri and Magdon-Ismail 2012). Therefore, we gave equal attention to both positive and **Negative degree** in this paper to conceptualise our data as signed networks. It is important to notice that a negative edge literately represents the attribute of the edge as a negative relationship or opposite synchronisation, but does not indicate a low or an absent interaction between nodes. Instead, two nodes could be highly interactive and have a strong relationship with a negative edge (Newman 2010). Let $A^{-}_{ij}$ denote the negative correlation identified in an adjacency matrix, then:
  5 $$ k^{-}_{i}=\sum\limits_{j}^{n} A^{-}_{ij}  $$
4. **Eigenvector centrality** can be seen as an extension of the degree centrality but shows consideration to the relative importance of a node’s neighbours. This centrality measure, firstly proposed by Bonacich (1987), defines centrality *e*_*i*_ as proportional to the sum of the centrality of neighbour nodes of *i*, let *κ*_1_ be the largest eigenvalue of matrix *A*, we have:
  6 $$ e_{i} = \frac{1}{\kappa_{1}}\sum\limits_{j} A_{ij} e_{j}  $$
5. A very useful centrality measure for depicting the relation between pairs of nodes is known as **Clustering coefficient** (CC), sometimes also referred to as transitivity. For each individual node, the CC is always defined as the local clustering coefficient, which represents the average probability that a pair of node *i*’s neighbours are also connected (Newman 2010).
  7 $$ c_{i} = \frac{number\quad of\quad pairs\quad of\quad i's\quad neighbour\quad that\quad are\quad connected}{number\quad of\quad pairs\quad of\quad i's\quad neigbhour}  $$
6. The last one is a fairly straightforward measure of node *i*’s neighbourhood condition. The **Average neighbour degree** (Ave. neigh. degree) measures the average number of degree that connected to *i*’s neighbours. Let *i* has *n* neighbours and their degree can be expressed as *k*_*j*_, then:
  8 $$ x_{i} = \frac{\sum_{j}^{n} k_{j}}{n}  $$

**Fig. 3:**
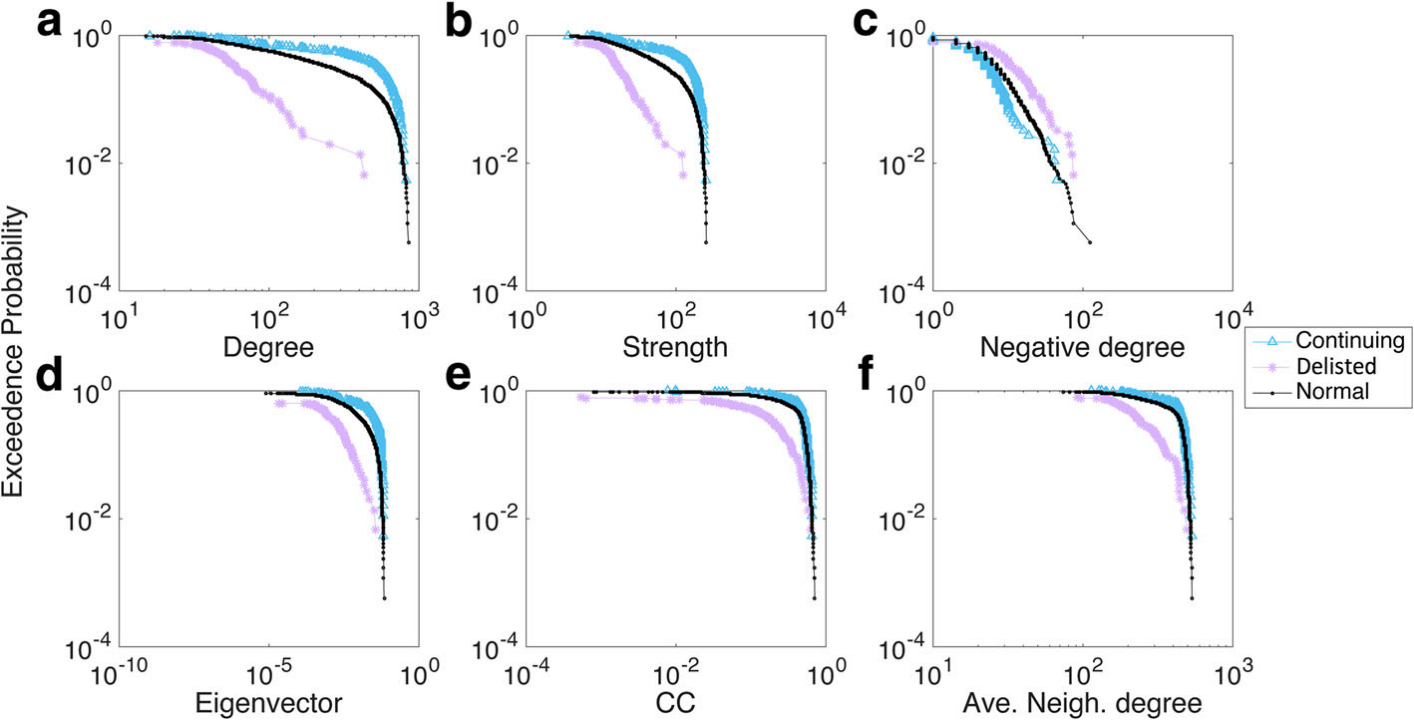
Distribution of the six network measures in 1988-1989. **a** Degree; **b** Strength; **c** Negative degree; **d** Eigenvector; **e** CC; **f** Ave. Neigh. degree

We calculated all six network measures for each stock in every stock group during the 40-year period. Using 1988-1989 as an example, Fig. 3a-f illustrates the exceedance probability distribution of network measures in the three groups. A clear gap existed between the Delisted group and the other two groups, indicating that the Delisted stocks behaved differently in terms of all six measures. However, the differences were not as easily spotted between the Continuing and Normal groups, except in the degree and strength distribution plots (Fig. 3a-b). We found it interesting that subplot (c) revealed a reverse order in the distribution of negative degree for a node, i.e., stocks from the Delisted group tended to have larger negative degrees when compared with stocks from the Continuing group, while Normal stocks fell in between.

**Fig. 4:**
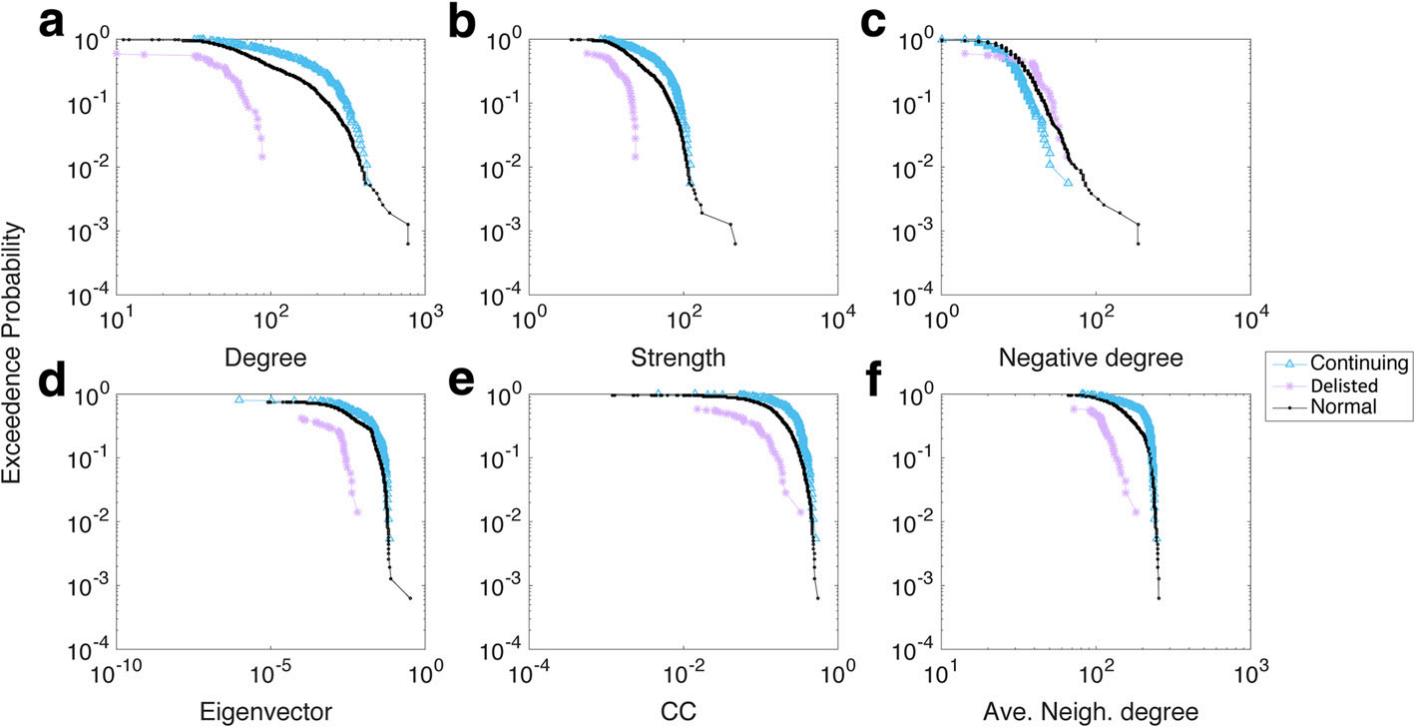
Distribution of the six network measures in 1993-1994. **a** Degree; **b** Strength; **c** Negative degree; **d** Eigenvector; **e** CC; **f** Ave. Neigh. degree

A similar tendency was found for other years, such as those seen from 1993 to 1994 (Fig. 4a-f). There, the negative degree distribution profile indicated some variations because the gaps among each group pair were not very obvious, and even some crossing and entanglement were found. However, the gaps between each pair of groups were generally clear and distinct, such as the significant difference noted in 2003-2004 (see Appendix 2). Thus, we confirmed that each group differed in terms of their network nodal features, thereby allowing us to use those differences as appropriate features when characterising the survivability performance of stocks within each group.

## Survivability and resilience characterisation

In this section, survivability analysis based on aforementioned network measures are presented. In order to study the possible relationship between stock survivability resilience and dynamic network measures, we constructed a model to characterise the different groups and explored the explanatory strength of each variable. The method applied here was selected as weighted multinomial logistic modelling. The particular reason for such selection is three-fold: First, we categorised all stocks into three nominal groups and that raises a problem of dealing with multi-class classification. Multinomial logistic regression is known to be suitable to handle dependent variable which has more than two levels. Second, because the populations of three groups were unbalanced in our data (a large portion of stocks are from the Normal group), we used penalised/weighted multinomial logistic regression to “re-balance” the groups by specifically assigning biased weights according to their actual number of observations. Third, as explained previously (Alaka et al. 2017), the logistic-based classifiers have been shown to possess high transparency in understanding of detailed parameters. Even though their accuracy may not be as excellent as other popular machine-learning classifiers, their capability to facilitate decomposition analysis is still outstanding. Last but not the least, we had to consider that the regression could only show how the variation in predictive variables co-occurs with variation in response. There is no cause-and-effect relationship guaranteed between survivability resilience and nodal interdependence just based on regression analysis (Montgomery et al. 2012).

### Weighted multinomial logistic modelling

Multinomial logistic models depict the relationship between response probabilities and all six predictors, node degree *k*_*i*_, node strength *s*_*i*_, negative degree $k^{-}_{i}$, eigenvector *e*_*i*_, cluster coefficient *c*_*i*_ and average neighbour degree *x*_*i*_. By their very nature, such models provide the estimated probability or odds of a target group against a reference group and, in our case, can be presented in the form as:

9 $$ ln \left(\frac{\alpha}{\gamma}\right)=A+B\times k + C\times s + D\times k^{-} + E\times e + F\times c + G\times x  $$

where *α* is the target group, *γ* is the reference group, *A* is the intercept term of the model and *B*, *C*, *D*, *E*, *F* and *G* are coefficients of the six covariates. Because we were more interested in the Delisted and Continuing groups as our targets, we used the Normal population as the reference group. Therefore, we modelled the first two against the Normal group and transformed the dependent variables into nominally distributed responses, where “1” represented the Delisted group, “2” was for the Continuing group, and “3” indicated stocks in the Normal group.

The data used to calibrate the models were network data from 1984-2012. The first seven years of networks, cross-referencing Fig. 1b, were not used due to their extremely unbalanced number in the Delisted group (very low number of observations) and the last five years, 2012-2017, were selected to be used as testing sets in later sections. This left 28 networks, from 1984 to 2012, for model training and calibration. From there, we gained a total of 55903 observations, among which 4875 were in the Delisted group; 5096, in the Continuing group; and the remaining 45932 stocks, in the Normal group.

Before starting the model training, it takes only a moment’s reflection to realise that apart from two special groups (Delisted and Continuing) the majority of the population would, of course, be in the Normal group. The class imbalance problem, if left untreated, could have potentially biased the estimated calibration results and lose accuracy due to different distributions of each class. Treatments for such issue have always been a topic in statistics and machine learning communities (Mosley 2013). There are several methods are claimed as effective such as over-sampling, under-sampling, synthetic minority over-sampling technique (SMOTE) (Chawla et al. 2002) and threshold-moving methods. Yet those methods have only been empirically observed as effective in most of the binary classifications, and a satisfactory solution for multi-class unbalance problem still needs investigation (Han et al. 2011). Here, we applied penalised/weighted models for two aspects of consideration: First, the over-sampling and under-sampling approaches would have required random deletions or duplicate tuples in groups, which would have involved unavoidable manipulation of the original data. It also would have been difficult to decide which of the majority and minority groups to be under- and over-sampled, respectively. Second, because we had decided on a fixed model type and were unwilling to manipulate tuple data, a good alternative was to assign weights to bias the model, thereby giving more attention to the minority group. Furthermore, by not manipulating the data, our choice provided a different perspective on the problem by adjusting the models per se.

Each stock can be modelled with a penalised weight determined by its class group during the fitting process. Given a series of multi-class as 1,2,3,... *i*,…*n* in total, the weight for class *i* can be determined as:

10 $$ w_{i}=\frac{\left(\sum_{i=1}^{n}N_{i}\right)/n}{N_{i}}  $$

where *N*_*i*_ is the number of observation in class *i*. In our case, the stocks in the Delisted group had a penalised weight of $\frac {55903/3}{4875} = 3.822$, while the weights of the Continuing group and Normal group were 3.657 and 0.401, respectively. One can see that the two minority groups eventually had relatively higher weights than the majority Normal group.

Table 4 lists the estimated coefficients and their standard errors for the log odds of two groups against the Normal group. The coefficients indicate the effects of the predictor variables on the log odds of being in one category versus the reference category. We can also notice one interesting observation that all of the signs for the coefficients estimated in the Delisted and Continuing groups were completely reverse. In other words, the different behaviour of the Delisted and Continuing stocks, in terms of network measures, could relate to reverse effects of the same variables. The standard errors for all predictor variables were rather small.

**Table 4:** Estimated coefficients and corresponding standard errors

	Intercept	Degree	Strength	Neg.degree	Eigenvector	CC	Ave.neighbour.degree
Coefficients							
Delisted/Normal	0.841	-0.047	0.140	0.003	-6.372	-5.184	0.003
Continuing/Normal	-0.421	0.003	-0.005	-0.003	1.346	3.805	-0.004
Standard Errors							
Delisted/Normal	0.0190	0.0013	0.0038	0.0008	0.0007	0.0155	0.0001
Continuing/Normal	0.0218	0.0007	0.0021	0.0009	0.0039	0.0939	0.0001

In addition, we tested the significance of the estimated coefficients. We firstly performed a two-tailed z test. Table 5 indicates that all estimated coefficients were very significant for estimation on both groups (very small values). Moreover, a Type III analysis of variance (ANOVA) was carried out to verify this result with an overall significance test on all variables. The test contains evaluation on likelihood-ratio chi-square statistic (LR Chisq) test and their significance p-value test. We can see from Table 6 that all variables were tested as “significant” in our modelling analysis.

**Table 5:** Two-tailed z test on significance level of estimations

	Intercept	Degree	Strength	Neg.degree	Eigenvector	CC	Ave.neighbour.degree
Two-tailed z test							
Delisted/Normal	0	0	0	1.136 ×10^−3^	0	0	0
Continuing/Normal	0	7.841 ×10^−6^	2.651 ×10^−2^	1.157 ×10^−4^	0	0	0

**Table 6:** Type III ANOVA test on likelihood-Ratio chi-square test and *p*-value test

	LR Chisq	Degree of Freedom	Pr(>Chisq)	Significance level^a^
Type III ANOVA				
Degree	1937.55	2	<2.2×10^−16^	***
Strength	1697.76	2	<2.2×10^−16^	***
Neg.degree	30.79	2	2.056 ×10^−7^	***
Eigenvector	65.61	2	5.656 ×10^−15^	***
CC	3078.99	2	<2.2×10^−16^	***
Ave.neighbour.degree	2979.82	2	<2.2×10^−16^	***

^a^Significance indicator: 0 ‘***’,0.001 ‘**’, 0.01 ‘*’, 0.05 ‘ ^*o*^’

Thus, we write:

11 $$ ln\left\{\frac{P(Delisted)}{P(Normal)}\right\}= 0.841 - 0.047 k + 0.140 s + 0.003 k^{-} - 6.372 e - 5.184 c + 0.003 x  $$

12 $$ ln\left\{\frac{P(Continuing)}{P(Normal)}\right\}= -0.421 + 0.003 k - 0.005 s - 0.003 k^{-} + 1.346 e + 3.805 c - 0.004 x  $$

where *P*(.) is the probability of being a particular category. Let *y*1 denotes *ln*(*Delisted/Normal*) and *y*2=*ln*(*Continuing/Normal*), then taking exponential on both sides of the equation, we have:

13 $$ \frac{P(Delisted)+P(Continuing)}{P(Normal)} = \frac{1-P(Normal)}{P(Normal)} = e^{y1} + e^{y2}  $$

therefore, we were able to calculate the probabilities of an observation being in each category as:

14 $$ P(Normal)=\frac{1}{1+e^{y1}+e^{y2}}   $$

15 $$ P(Delisted)=\frac{e^{y1}}{1+e^{y1}+e^{y2}}   $$

16 $$ P(Continuing)=\frac{e^{y2}}{1+e^{y1}+e^{y2}}   $$

Here, we obtained Eqs. (14)-(16) as quantitative assessments of the survivability resilience of stocks. For a given stock with corresponding network measures, three probabilities were associated with its calculation of survivability resilience, and the final categorisation of such stock depended upon the most likelihood (largest probability) of being in each different group.

To investigate further, we performed an analysis of deviance to test the explanatory strength of interactive predictors. As shown in Table 7, node degree, average neighbour degree, and strength were the first three influential terms that contributed the most to the reduction of residual deviance, i.e., 10919.2 from *k*_*i*_, 2979.8 from *x*_*i*_, and 2777.2 from *s*_*i*_. This indicated that these three degree-based measures contributed more in terms of reducing deviance to the resilient response probability when compared with other centrality measures.

**Table 7:** Analysis of deviance

Variables	Deviance	Residual deviance
Intercept		122831
Intercept+*k*	10919	111912
Intercept+*k*+*s*	2777	109135
Intercept+*k*+*s*+ *k*^−^	1520	107615
Intercept+*k*+*s*+ *k*^−^+*e*	17	107598
Intercept+*k*+*s*+ *k*^−^+*e*+*c*	737	106861
Intercept+*k*+*s*+ *k*^−^+*e*+*c*+*x*	2980	103881

**Fig. 5:**
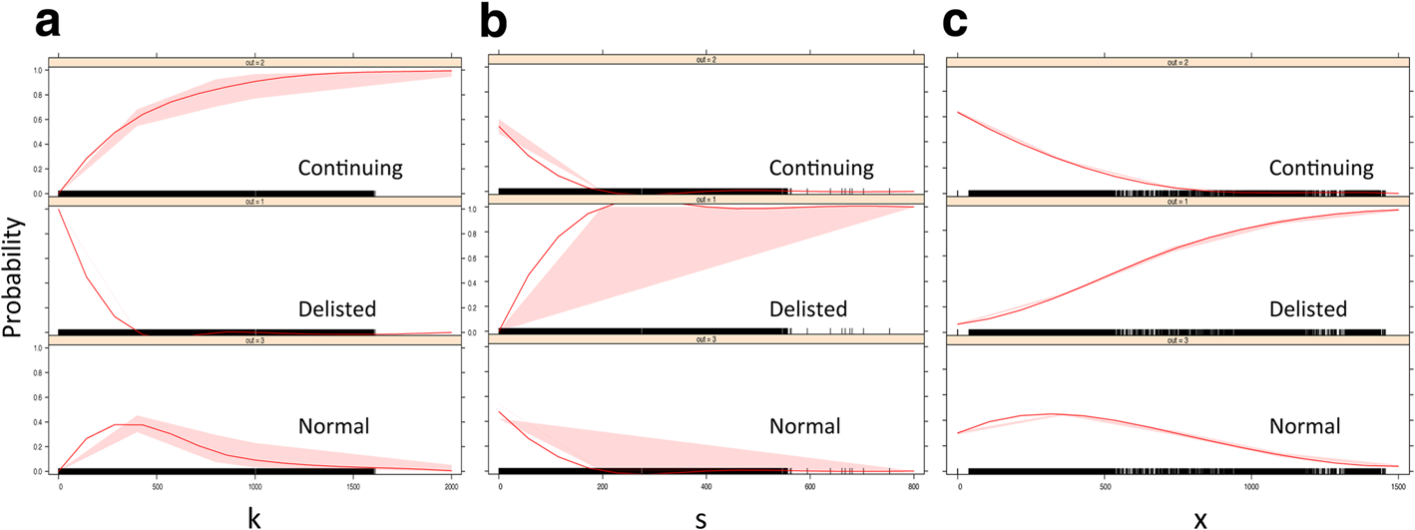
Effect displays analysis of the the three degree-based measures on all stock groups. **a** degree; **b** node strength; **c** average neighbour degree

Figure 5 shows the effect displays of these three degree-based variables in terms of quantified probability for all three groups. In subplot (a) to (c), we can see the probability of being modelled as Delisted was relatively sensitive to the changes in these three variables (probability value varies in full range from zero to one). In contrast, the sensitivity associated with Normal group fluctuated within a small range. For example, no matter how much drop or raise occurred in these three variables, the maximum probability of being modelled as Normal members were always less than 0.5. Meanwhile, their effects on modelling probability for Continuing group seemed to be in the middle of the former two.

### Model testing

**Fig. 6:**
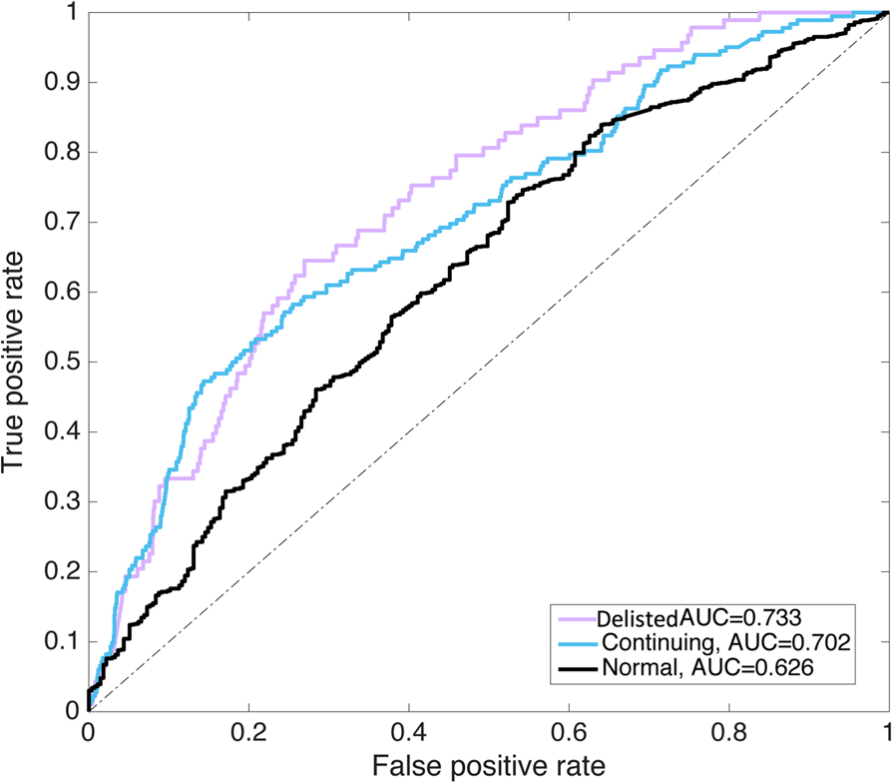
One-vs-rest ROC analysis of three groups of stocks in 2012-2013

The multinomial logistics model was validated and tested using network data from the last five years of observation, 2012-2017. Taking 2012-2013 as an example, Fig. 6 depicts the Receiver Operating Characteristic (ROC) curve analysis of the model performance when predicting the survivability resilience of the stocks during that time period. Because the ROC curve is normally used for binary classifiers, we plotted a one-vs-rest ROC curve for each class. The Area Under Curve (AUC) was adopted as an illustrative indicator that quantitatively demonstrated the diagnostic ability of the model. As shown in the figure, model performance with regard to predicting Delisted (AUC =0.733) and Continuing (AUC =0.702) stocks was relatively higher than when it was applied for predicting stocks from the Normal group (AUC =0.626). This might have resulted from the range of dynamic behaviour of network measures associated with stocks from different groups, which meant that the uniqueness of nodal interdependence from stocks in the Delisted and Continuing groups could potentially be more abnormal.

**Fig. 7:**
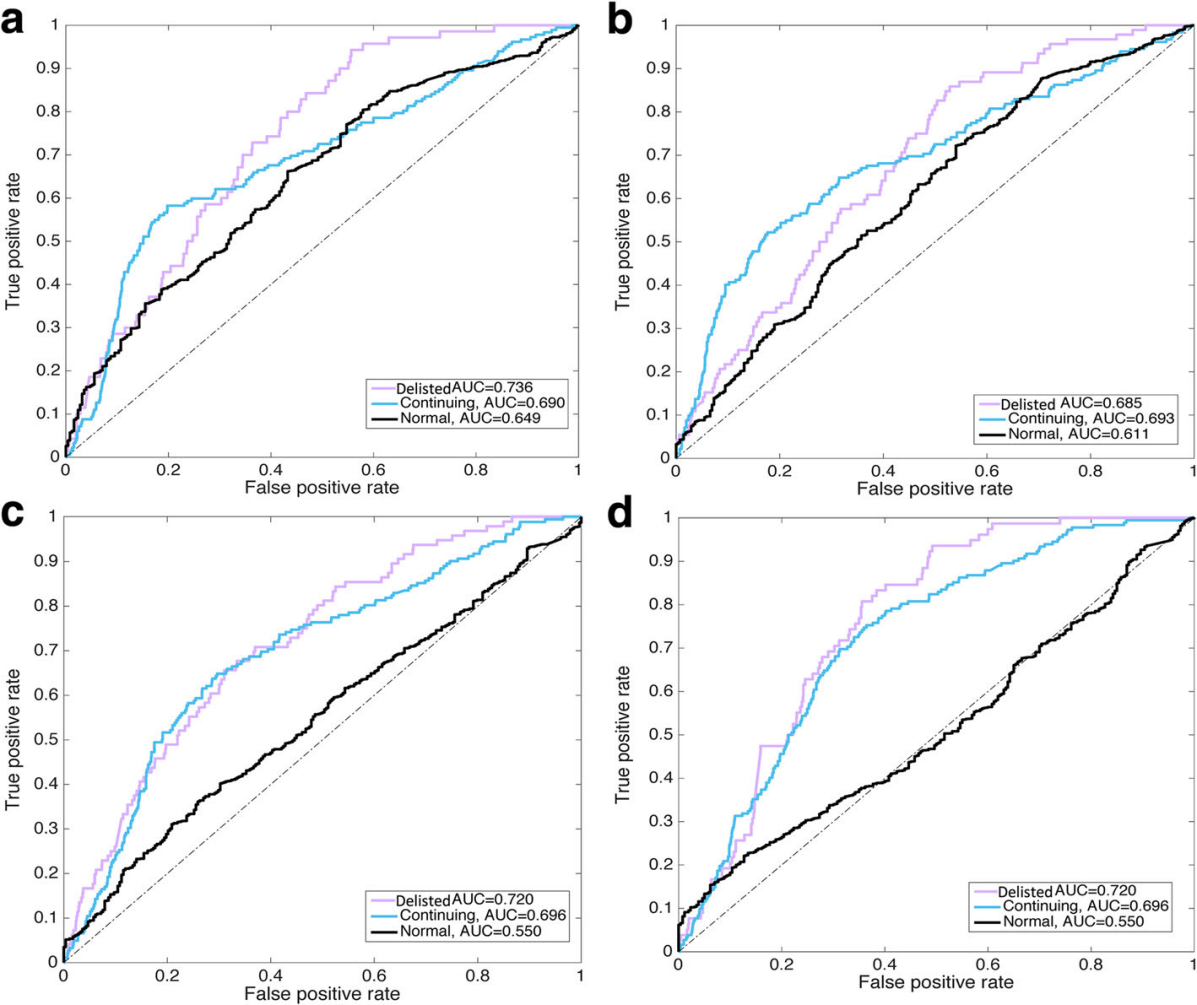
ROC analysis of three groups of stocks in **a** 2013-2014, **b** 2014-2015, **c** 2015-2016 and **d** 2016-2017

By observing ROC plots in Fig. 7, it even further enhances such interpretation as the AUC values for the Delisted and Continuing groups remained relatively stable around 0.69 to 0.74, while the AUC for Normal group gradually decreased from 0.649 to 0.550, indicating an increasing difficulty to accurately identify stocks with normal nodal behaviour. However, that might have been more achievable if one considered the rationale behind the network measures of these interactive nodes. That is, the continuing stocks would very likely still exist in the near future and, because they were becoming more influential in the core area of the market, then more stocks would tend to correlate with them. This would result in a growing interdependence degree within the networks. Of course, such growth would be heavily subjected to dynamic changes and shifts as the market evolved. However, stocks from the Normal group might also tend to waver between states of failure and continuation, therefore making their accurate identification fairly difficult. Coincidentally, this matches with the sensitivity insights we found in the aforementioned effect display tests.

**Fig. 8:**
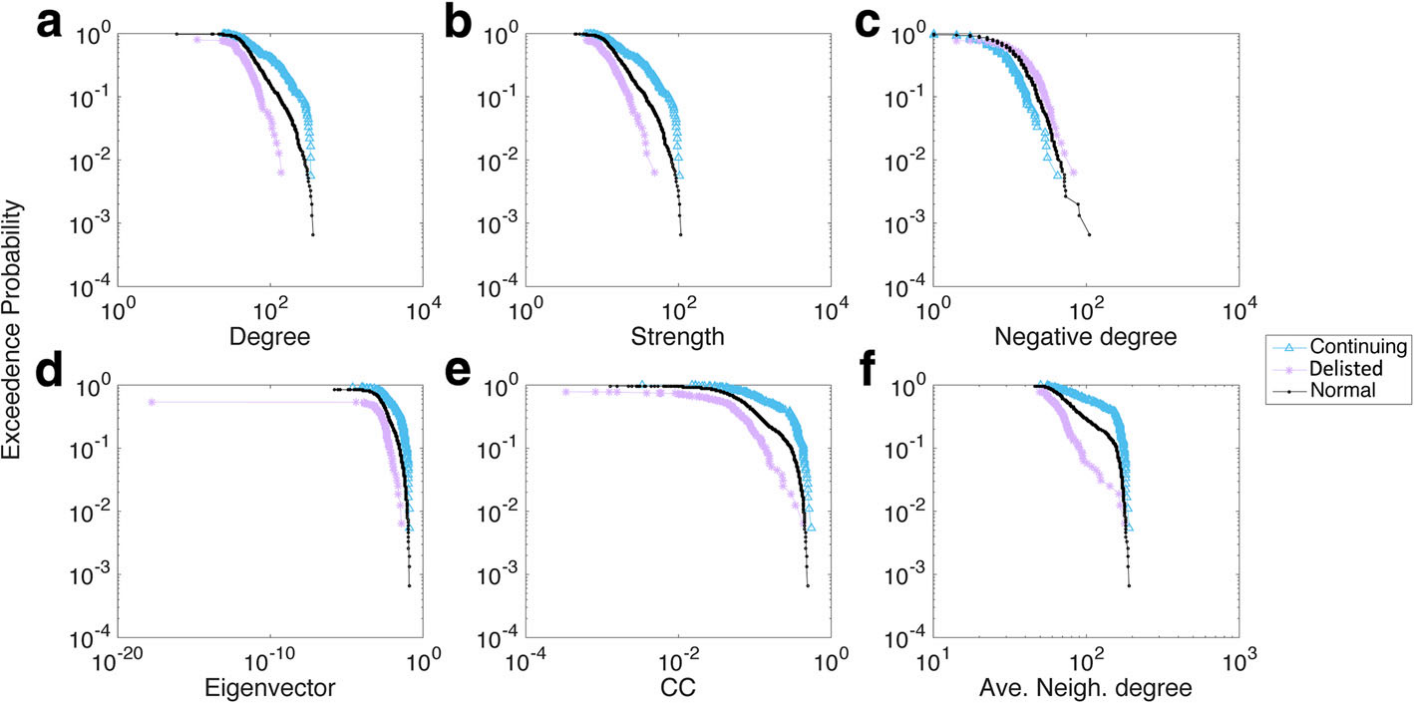
Distribution of the six network measures in 2003-2004. **a** Degree; **b** Stength; **c** Negative degree; **d** Eigenvector; **e** CC; **f** Ave. Neigh. degree

## Conclusion

We addressed the issue of characterising a stock’s survivability resilience in terms of bankruptcy prediction, using interdependent correlation-based networks. Relying upon big financial market data, we constructed these weighted, signed, and temporal networks based on correlations between stock pairs according to their daily adjusted closing prices. As a first step in exploring the dynamically evolving topology of the networks, we identified six suitable measures of network centrality and characterised different stock behaviours in terms of survivability. To maintain model transparency for each variable, we used those centrality measures as predictor variables in a weighted multinomial logistics model and conducted the further statistical analysis.

**Fig. 9:**
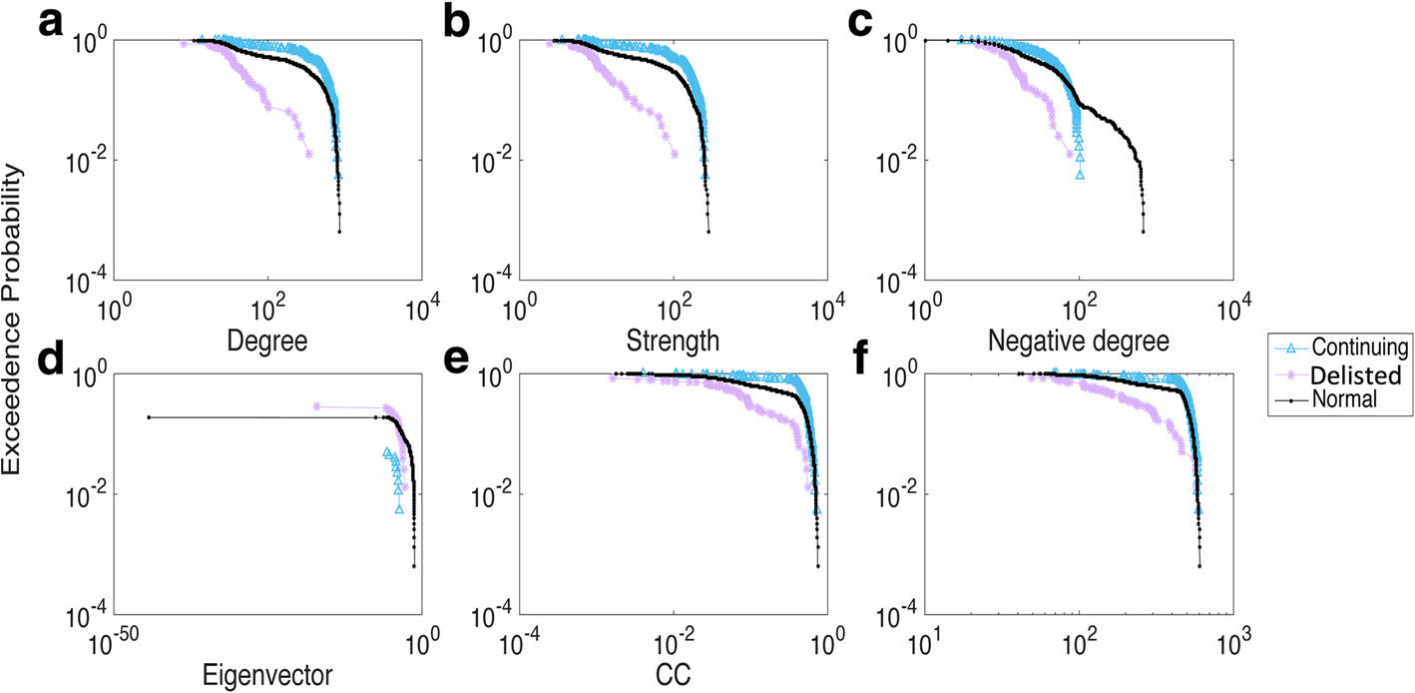
Distribution of the six network measures in 2016-2017. **a** Degree; **b** Stength; **c** Negative degree; **d** Eigenvector; **e** CC; **f** Ave. Neigh. degree

This study produced three main findings: First, the market, counterintuitively, does not constantly expand exponentially if one considers yearly dynamic “fission-fusion” shifting. Instead, major fluctuations occur, possibly because the market responds to unexpected external stimuli by dynamically adjusting nodal interdependence. Second, centrality-based network measures were useful predictive variables when characterising failed or resilient stocks because those measures can effectively capture the abnormal behaviour of such stocks. Finally, the results of analysis and model testing suggested that degree-based measures, including node degree, average neighbour degree, and node strength, could be applied as descriptive parameters for characterising the survivability resilience of equities in the London Stock Exchange. However, the effect of variables and AUC values obtained from the Normal group indicated that stocks from this group were more difficult to depict.

This study provides insights for quantitatively assessing and modelling the survivability resilience of stocks in the London Stock Exchange. We propose a new perspective that utilises statistical topology measures to assess company resilience in interdependent complex networks. Future research could focus on higher-fidelity characterisations and representations within such complex, dynamic, and temporally evolving systems, and comparative studies on different network construction methods, data treatment algorithms, and modelling techniques could be carried out as well. The findings are useful for identifying early signals of firms in potential financial difficulties, which can help for various decision- and policy-makers such as investors, creditors, and managers.

## Appendix 1: Partial correlation coefficients with excess return

Taking 2016-2017 as an example, we constructed the network with Partial correlation coefficient method. The benchmark for calculating excess return was SPDR S&P 500 ETF index, which collected with a same periodicity within 2016-2017. The correlation matrix was obtained by applying Partial correlation coefficient function in MATLAB (Mathworks) with the residuals against the benchmark. The percentage of the positive coefficient was around 62.26% (dropping from 78.63% from Table 3) with negative ones around 37.74%. This shows an interesting comparative result as the portion of negative correlations greatly increased.

## Appendix 2: Distributions of six network measures in 2003-2004 and 2016-2017

Figures 8 and 9 illustrate the distribution of all six network measures with respect to different groups of companies in 2003-2004 and 2016-2017. The aforementioned gaps were still obvious in those two later years. Because these differences in distribution remained throughout the observation period, one might infer that they were a general feature associated with each group rather than being simply random outcomes.

## Abbreviations

AUC: Area under curve
CC: Clustering coefficient
ROC: Receiver operating characteristic
